# Overexpressing the H‐protein of the glycine cleavage system increases biomass yield in glasshouse and field‐grown transgenic tobacco plants

**DOI:** 10.1111/pbi.12953

**Published:** 2018-07-22

**Authors:** Patricia E. López‐Calcagno, Stuart Fisk, Kenny L. Brown, Simon E. Bull, Paul F. South, Christine A. Raines

**Affiliations:** ^1^ School of Biological Sciences University of Essex Colchester UK; ^2^ Global Change and Photosynthesis Research Unit United States Department of Agriculture/Agricultural Research Service Urbana IL USA; ^3^ Carl R. Woese Institute for Genomic Biology University of Illinois Urbana IL USA; ^4^ Present address: Molecular Plant Breeding Institute of Agricultural Sciences ETH Zürich 8092 Zürich Switzerland

**Keywords:** photosynthesis, photorespiration, glycine decarboxylase H‐protein, chlorophyll fluorescence imaging, transgenic, yield, lipoylation

## Abstract

Photorespiration is essential for C3 plants, enabling oxygenic photosynthesis through the scavenging of 2‐phosphoglycolate. Previous studies have demonstrated that overexpression of the L‐ and H‐proteins of the photorespiratory glycine cleavage system results in an increase in photosynthesis and growth in *Arabidopsis thaliana*. Here, we present evidence that under controlled environment conditions an increase in biomass is evident in tobacco plants overexpressing the H‐protein. Importantly, the work in this paper provides a clear demonstration of the potential of this manipulation in tobacco grown in field conditions, in two separate seasons. We also demonstrate the importance of targeted overexpression of the H‐protein using the leaf‐specific promoter ST‐LS1. Although increases in the H‐protein driven by this promoter have a positive impact on biomass, higher levels of overexpression of this protein driven by the constitutive CaMV 35S promoter result in a reduction in the growth of the plants. Furthermore in these constitutive overexpressor plants, carbon allocation between soluble carbohydrates and starch is altered, as is the protein lipoylation of the enzymes pyruvate dehydrogenase and alpha‐ketoglutarate complexes. Our data provide a clear demonstration of the positive effects of overexpression of the H‐protein to improve yield under field conditions.

## Introduction

The photorespiratory pathway is a major route for the flow of carbon in the biosphere and is intrinsically linked to photosynthetic carbon assimilation through the Calvin–Benson–Bassham (CB) cycle. When ribulose 1,5‐bisphosphate carboxylase–oxygenase (Rubisco) fixes O_2_, instead of CO_2_, to ribulose 1,5‐bisphosphate (RuBP) in an oxygenation reaction, one molecule each of 3‐phosphoglycerate (3PGA) and of 2‐phosphoglycolate (2PG) are produced. The photorespiratory pathway scavenges this 2PG and releases CO_2_ following reactions that occur in the chloroplasts, mitochondria, peroxisomes and cytosol.

Although indispensable for C3 photosynthesis, photorespiration is estimated to reduce the theoretically attainable efficiency of the process by 48% (assuming a carbon dioxide concentration of 380 ppm and a temperature of 30 °C) which represents a potential limitation on carbon gain in C3 plants and, ultimately, crop yields (Walker *et al*., 2016; Zhu *et al*., 2008). Manipulations aimed at blocking the photorespiratory pathway, however, resulted in a number of detrimental effects on plant growth and development (Brisson *et al*., 1998; Rachmilevitch *et al*., 2004; Walker *et al*., 2016). In an attempt to overcome the potentially negative impact of reducing photorespiratory flux, while improving carbon assimilation by increasing CO_2_ concentration in the chloroplast and reducing the energy requirement of the process, photorespiratory bypasses have been engineered with very promising outcomes (Carvalho *et al*., 2011; Kebeish *et al*., 2007; Maier *et al*., 2012; Peterhansel *et al*., 2013). For example, *Arabidopsis thaliana* plants expressing two of the proposed bypasses displayed a 30%–50% increase in biomass (Kebeish *et al*., 2007; Maier *et al*., 2012). Facilitating photorespiratory carbon flow through overexpression of subunits of the glycine cleavage system (GCS) appears to be another viable approach for improving photorespiration [reviewed in Timm *et al*. (2016)]. In plants, the GCS (also named glycine decarboxylase or glycine dehydrogenase system) produces CO_2_ from the photorespiratory process and, together with serine hydroxymethyltransferase, is responsible for the interconversion of glycine and serine, an essential and ubiquitous step of both photorespiration and primary metabolism (Bauwe and Kolukisaoglu, 2003). The GCS comprises four proteins, three enzymes (P‐protein, T‐protein and L‐protein) and a small lipoylated protein known as H‐protein, which interacts with the others by commuting from one enzyme to the other. Although the H‐protein has no catalytic activity, it acts as a substrate for the P‐, T‐ and L‐proteins and has been demonstrated, *in vitro*, to be capable of enhancing the activity of the GCS (Hasse *et al*., 2009). Furthermore, overexpression of this protein in *A. thaliana* has led to increases in biomass, maximum relative electron transport rate, light saturation point and net CO_2_ uptake (Timm *et al*., 2012). In a similar manner, overexpression of the L‐protein has also been shown to increase plant growth and improve photosynthesis by facilitating photorespiratory carbon flow through the GCS (Timm *et al*., 2015). Additionally, overexpression of the H‐protein in combination with CB cycle enzymes sedoheptulose‐1,7‐bisphosphatase (SBPase) and fructose‐1,6‐bisphosphate aldolase (FBPA) has been shown to further improve plant growth (Simkin *et al*., 2017).

The aim of the work reported in this paper was to investigate the effect of overexpression of the H‐protein on plant growth and performance in the model crop species *Nicotiana tabacum* cv. Petite Havana (tobacco), under both controlled environment and field conditions.

## Results

### Production and molecular characterization of plants overexpressing H‐protein

**Figure 1:**
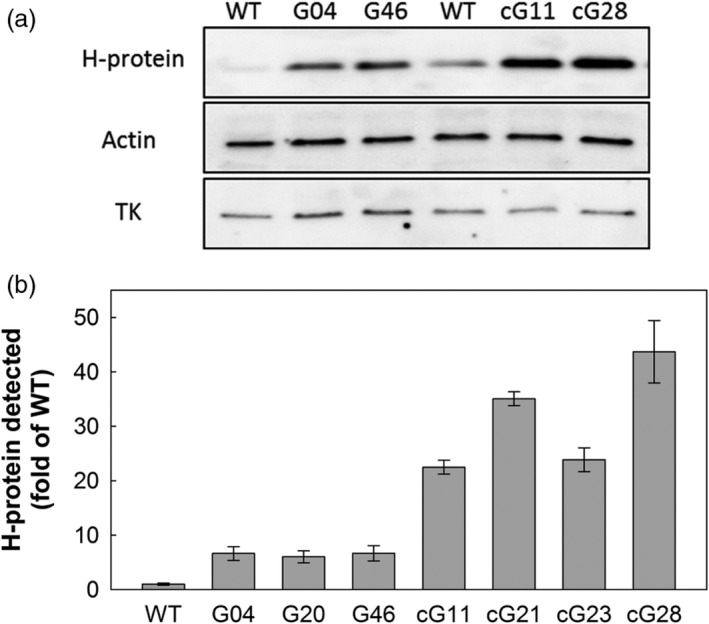
Expression of the H‐protein in transgenic plants. H‐protein expression in the leaf (a) of WT, ST‐LS1::H OX lines (G04 and G46) and 35S::H OX lines (cG11 and cG28). Actin and TK were used as loading controls; approximately 8 μg of total protein were loaded per well. (b) Relative H‐protein signal in immunoblots between WT, ST‐LS1::H and 35S::H OX (all samples were normalized to transketolase signal, immunoblots in Figure S2).

Transgenic tobacco plants were produced following *Agrobacterium*‐mediated transformation. To avoid cosuppression, the full‐length *A. thaliana* H‐protein cDNA (AT2G35370) was used. Two overexpression constructs were assembled using Golden Gate cloning technology (Engler *et al*., 2008, 2009). The first contained the leaf‐specific and light‐regulated, ST‐LS1, promoter from potato (Stockhaus *et al*., 1989; Timm *et al*., 2012) and the second contained the *CaMV* 35S constitutive promoter known to produce high levels of expression. These promoters were used to drive the transcription of the full‐length *A. thaliana* H‐protein cDNA (AT2G35370) and contained the commonly used HSP terminator sequence (Nagaya *et al*., 2010) (Figure S1). Five independent lines (each containing a single T‐DNA insertion) for each construct were allowed to self‐fertilized until T2 plants were produced. Overexpression of the H‐protein transgene in leaf tissue was confirmed in all generations using qRT‐PCR and immunoblotting. Based on transgene expression levels, three homozygous ST‐LS1::H lines (G04, G20 and G46), as well as two azygous (null mutants for T‐DNA) control lines derived from G04 and G20, termed aG04 and aG20, were selected for further analysis. Only one homozygous 35S::H OX line was identified (cG28), and therefore, three T2 hemizygous lines (cG11, cG21, cG23) with increased transgene expression were also selected. Successful overexpression of the H‐protein in leaf tissue was confirmed in experimental plants by immunoblotting, and a threefold to sevenfold increase in H‐protein for 35S::H OX compared to the ST‐LS1::H OX plants was detected (Figures 1, S2 and S3a). Additional immunoblot analyses for both the ST‐LS1::H and the 35S::H OX lines also indicated that there was no change in the levels of the other three proteins in the GCS (L, P and T) in any of our transgenic lines (Figure S2).

### Excess H‐protein negatively affects early growth in tobacco

**Figure 2:**
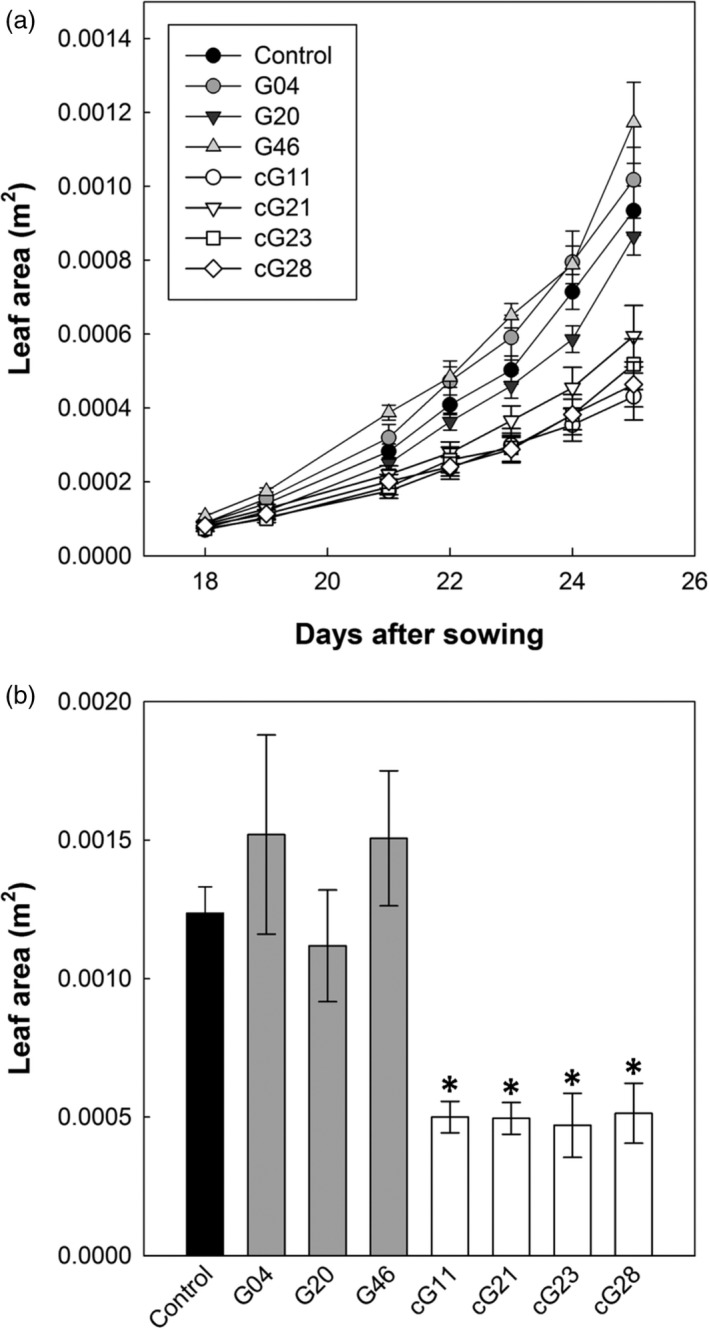
Overexpression of the H‐protein impacts on seedling leaf area. Plants were germinated and grown in a controlled environment growth room (22 °C, 16‐h light, 8‐h dark cycle) for 26 days and seedling leaf area determined. (a) Seedling growth over an 8‐day period. Control group (black circles), ST‐LS1::H protein overexpressors (grey circles), 35S::H protein overexpressors (white circles). (b) Seedling leaf area 26 day after sowing. Mean values ± SE are indicated, *n* = 5–12 plants per line. Control group represent both WT and azygous plants. Asterisks indicate significance between OX lines and control group using ANOVA with Tukey's post hoc test, *P < 0.05.

The ST‐LS1::H and 35S::H OX lines were grown in controlled environment conditions (16 h light, 8 h dark, at 22 °C) for 26 days and leaf area determined to assess growth. In addition, chlorophyll fluorescence (CF) imaging was used to examine the quantum efficiency of photosystem II (PSII) photochemistry (*F*_*q*_ *′/F*_*m*_ *′)* (Baker, 2008; Murchie and Lawson, 2013). No significant differences in *F*_q_ *′/F*_m_ *′* were detected when comparing control and either group of H‐protein overexpressing plants (data not shown). In contrast, differences in the leaf area became visibly apparent as early as 14 days after planting. The ST‐LS1::H OX seedlings had leaf areas either similar to or greater than (up to 26%) the control group 26 days after sowing (DAS). In contrast, the 35S::H OX lines displayed a significant reduction in leaf area throughout the early growth phase and, by 26 DAS, reached only 50% of control leaf area (Figure 2). This is consistent with changes in the relative growth rates (RGR) measured as variations in plant leaf area over time [RGR = [ln(LAf) − ln(LAi)]/(tf − ti)]. The smaller 35S::H OX plants had significantly lower RGR in four of the six time periods measured and did not differ from the control group on the other two periods. Contrastingly, the ST‐LS1::H OX plants had more similar RGR to the control group, significantly exceeding it over three time periods and only being significantly lower in day 24 after sowing (Figure S4).

### Overexpression of H‐protein in tobacco alters carbohydrates profile and protein lipoylation

**Figure 3:**
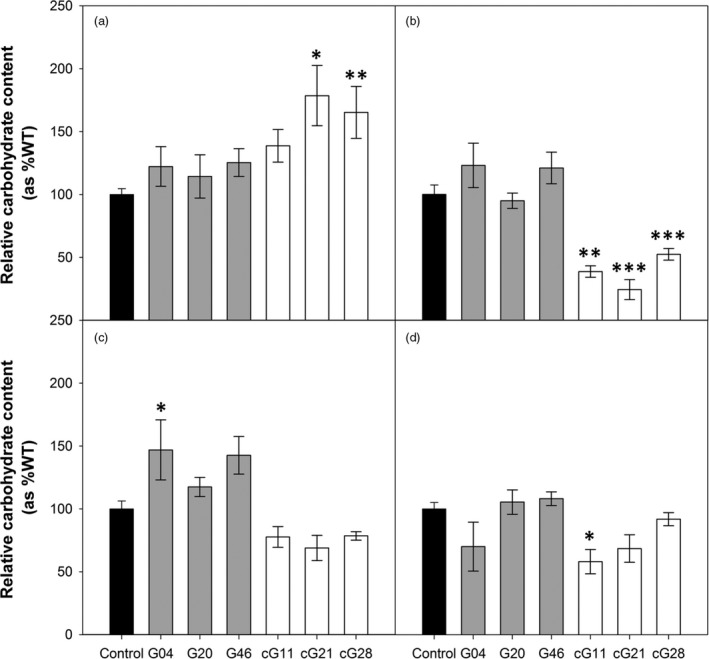
Overexpression of H‐protein can affect leaf carbohydrate pools. Carbohydrate contents in the youngest fully expanded leaf of 5‐week‐old glasshouse‐grown plants. (a) Starch, (b) glucose, (c) fructose, (d) sucrose. Mean values ± SE are indicated, *n* = 5 per line. Control group represent both WT and azygous plants. Asterisks indicate significance between OX lines and control group using ANOVA with Tukey's post hoc test, *P < 0.05, **P < 0.01, ***P < 0.005.

Leaf starch and soluble sugars (glucose, fructose and sucrose) content were determined to investigate whether carbohydrate profile is associated with the reduced leaf area phenotype observed in the 35S::H OX plants (Figure 3). Although the total amount of carbohydrates measured in the transgenic lines (in hexoses equivalent) was not significantly different to the control plants, the distribution of this carbon between the soluble and insoluble pools changed significantly in the 35S::H OX. These plants displayed reduced amounts of soluble sugars and a significant increase in accumulation of starch. In contrast, the ST‐LS1::H OX plants showed no significant differences for carbohydrate allocation at mid‐day.

H‐protein lipoylation is an essential post‐translational modification indispensable for the activity of the GCS. To examine protein lipoylation in our H‐protein overexpressors, immunoblots recognizing the lipoic acid (LA) group were performed using leaf and root tissue from both the ST‐LS1::H and the 35S::H OX plants. The LA signal corresponding to the H‐protein in leaf tissue of both the ST‐LS1::H and the 35S::H OX plants was higher than in the control plants (Figure S3a) which is in keeping with higher amounts of this protein in these transgenic plants. Interestingly, although the H‐protein amount (as reflected by the H‐protein antibody signal) in the leaves of the ST‐LS1::H OX plants is substantially less than the 35S::H OX plants, the lipoylation signal indicates a similar amount of lipoylated H‐protein in the leaves of ST‐LS1::H and the 35S::H OX plants.

Increased amounts of the lipoylated form of the H‐protein in roots were found only in the 35S::H OX lines (Figure S3b). In root tissue, the LA signal of pyruvate dehydrogenase (PDH) E2 and α‐ketoglutarate dehydrogenase (KGDH) E2 was reduced in the 35S::H OX lines when compared to the control. This change in the lipoylation of these enzymes was not seen in the ST‐LS1::H OX lines. In leaves, no major changes were evident in the LA signal corresponding to PDH and KGDH in either ST‐LS1::H OX or 35S::H OX.

Due to the negative impact in growth, together with the changes observed in lipoylation of root proteins, and leaf carbohydrate allocation in the 35S::H OX plants, only the ST‐LS1::H OX plants were studied further.

### Increased H‐protein results in increased biomass under glasshouse and field conditions

**Figure 4:**
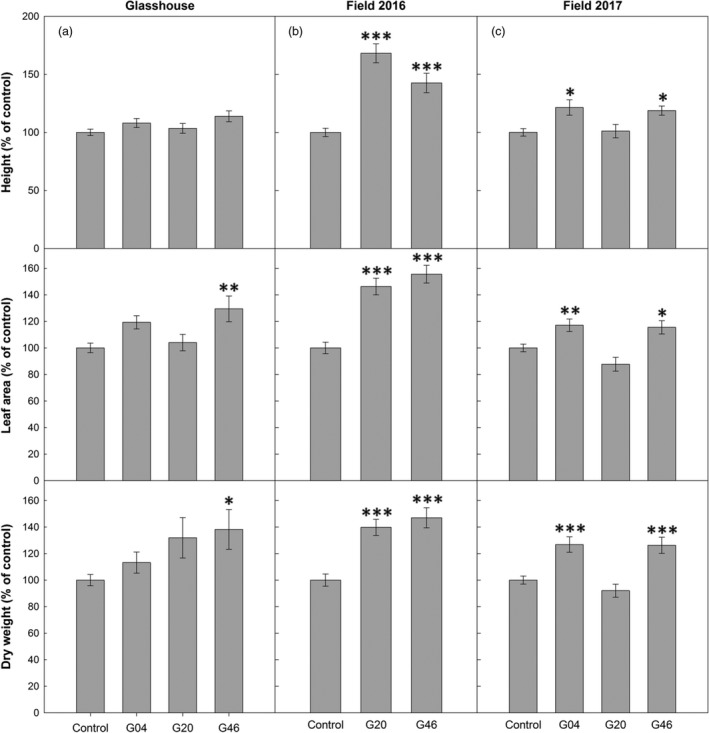
Overexpression of the H‐protein of the glycine cleavage system increases plant growth. (a) Forty‐day‐old glasshouse‐grown tobacco plants; (b) forty‐day‐old 2016 field‐grown plants. (Plants were germinated and grown in glasshouse conditions for 26 days and then allowed to grow in the field in summer 2016 for 14 days.); (c) Fifty‐six‐day‐old 2017 field‐grown plants. (Plants were germinated and grown in glasshouse conditions for 26 days and then allowed to grow in the field in summer 2017 for 30 days.) Plant height, leaf area and total above‐ground biomass dry weight are displayed. Control group represent both WT and azygous plants. Mean ± SE presented. Plants per line: (a) *n* = 4–6, (b) *n* = 6, (c) *n* = 24. Asterisks indicate significance between OX lines and control group using ANOVA with Tukey's post hoc test, *P < 0.05, **P < 0.005, ***P < 0.0005.

The ST‐LS1::H OX and control plants were grown in controlled environmental chambers and glasshouse conditions for approximately 6 weeks, at which point final biomass was determined. Our results showed the ST‐LS1::H OX plants displayed increased mean height, leaf number, leaf area and dry weight, leading to final above‐ground mean biomass increases of 13%–38% of control plants. Line G46 was significantly larger than the controls in leaf number, leaf area and dry stem and leaf biomass (Figures 4a and S5a,b).

To test whether the increases in biomass observed in these transgenic lines under glasshouse conditions could be reproduced in a field environment, the growth of these plants was evaluated in field experiments in two consecutive years under field conditions. In 2016, a small‐scale replicated control experiment was carried out using lines G20 and G46 (Figure S6a). Plants were germinated and grown as described in Figure 2 under controlled conditions for 25 days before being moved to the field. After 14 days in the field, plants were harvested and assessed per the glasshouse experiment. These data revealed a significant increase in biomass for both lines compared to the control plants (Figures 4b and S5c). This was reflected in increased number of leaves, height, total leaf area and a 40%–47% increase in total dry above‐ground biomass. In 2017, a larger scale, randomized block design field experiment was carried out using three independent lines (G04, G20, G46) (Figure S6b,c). Plants were grown from seed in the glasshouse for 33 days and then moved to the field and allowed to grow for 24 days before harvesting, at which point plants had started flowering. A significant increase in biomass was observed in these plants (Figures 4c and S5d). Although line G20 did not outperform the control group as observed the previous year, both line G04 and G46 displayed significant increases of at least 18%, 15% and 26% in height, leaf area and total dry above‐ground biomass, respectively.

### Photosynthetic CO_2_ assimilation rates are increased in ST‐LS1::H OX transgenic lines

**Figure 5:**
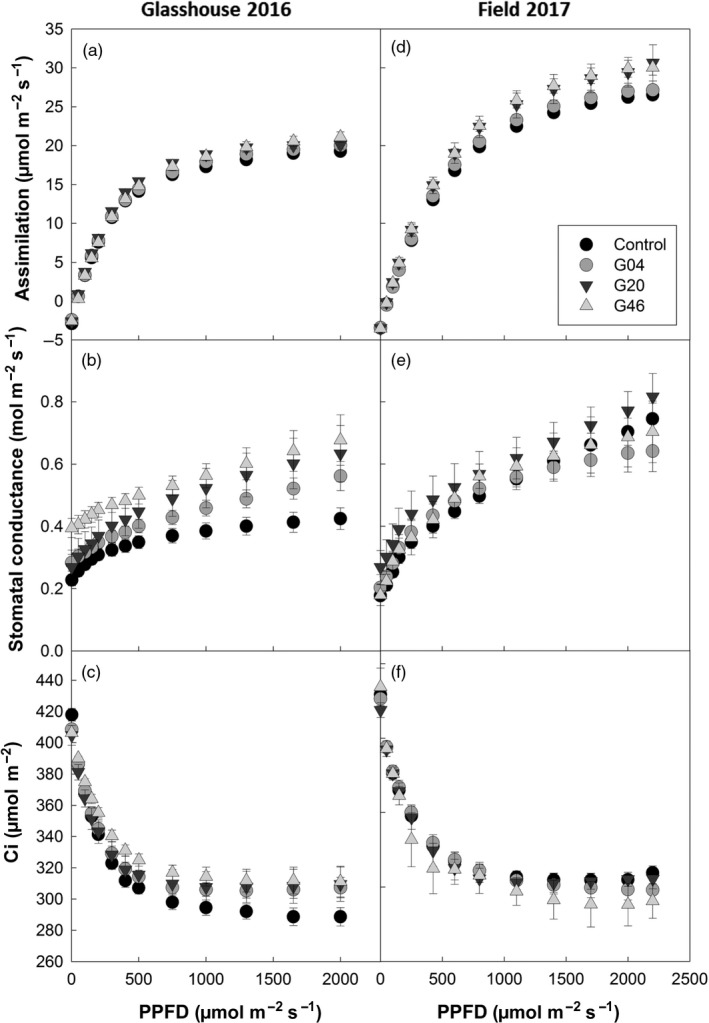
Overexpression of the H‐protein of the glycine cleavage system can increase CO_2_ assimilation and stomatal conductance. Photosynthesis as a function of light intensity (PPFD) of the plants described in Figures 4 and 5, under glasshouse conditions (a–c) and 2017 field conditions (d–f). (a, d) CO_2_ assimilation; (b, c) stomatal conductance; and (c, f) internal CO_2_ concentration (C_i_) *n* = 5–7 per line. Control represents a mix of WT and azygous plants.

To investigate whether the increases in biomass observed in the ST‐LS1::H OX were linked to increases in photosynthesis, CO_2_ assimilation (*A*) was measured in response to light intensity (*Q*). Photosynthetic rates increased in ST‐LS1::H OX plants grown in glasshouse and field conditions compared to controls (Figure 5). These differences in assimilation were most evident at higher light intensities, displaying higher means in all lines and were significantly increased (*P* < 0.05) in line G46 for a light intensity of 2000 μmol/m^2^/s in glasshouse‐grown plants, and for light intensities above 800 μmol/m^2^/s in field‐grown plants. Interestingly, under glasshouse conditions, these plants also displayed elevated stomatal conductance rates (*g*_s_) (only statistically significant in line G46) and the average internal CO_2_ concentration (C_i_) for each light point was higher than in the controls. This behaviour was not observed under field conditions, where lines G04 and G46 displayed either no changes or lower averages in conductance when compared to controls. Line G20, which in the 2017 field season did not display an increase in biomass compared to the control, maintained higher levels of *g*_s_ compared to the control, similar to that of the glasshouse‐grown plants.

### ST‐LS1::H OX transgenic lines have reduced damage to PSII caused by high photorespiratory conditions

**Figure 6:**
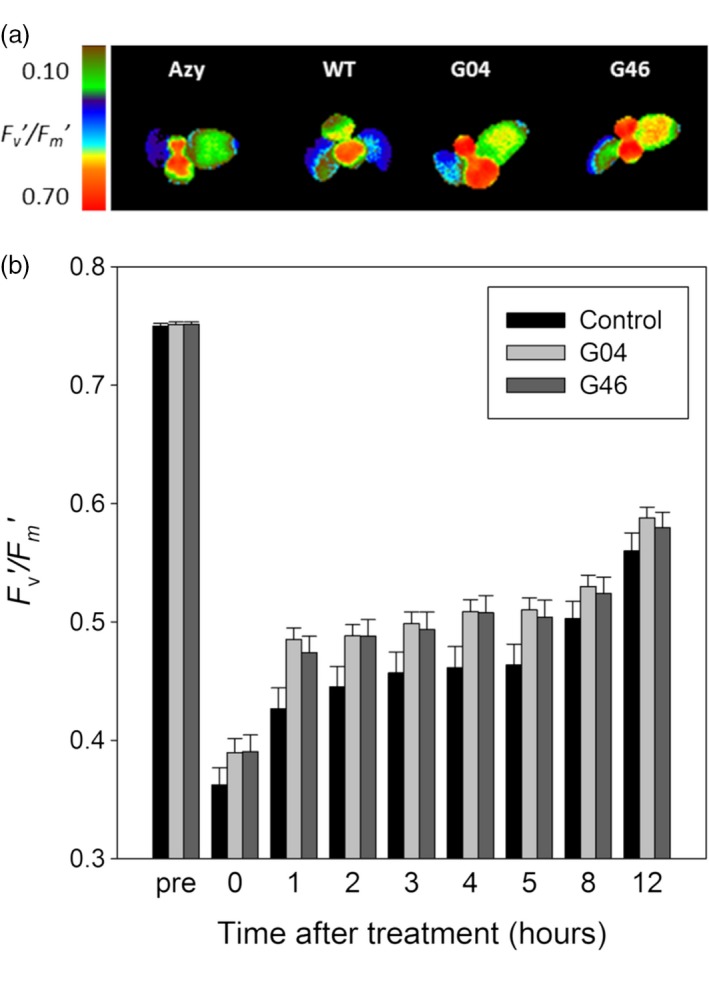
ST‐LS1::H OX plants experience less PSII damage under high photorespiratory conditions. Plants germinated and grown for 9 days in MS plates under controlled environment growth room (23 °C day, 18 °C night, 16‐h light, 8‐h dark cycle), were exposed to 24 h of high photorespiratory conditions (low CO_2_ and continuous high light) and then returned to normal growth conditions. *F*_v_′*/F*_m_′ measurements were taken before and after treatment, and every hour during the recovery process. (a) *F*_v_ *′/F*_m_ *′* of controls (azygous and WT plants), G04 and G46 lines after 24 h low CO_2_ under continuous light treatment. Scale bar represents an *F*_v_ *′/F*_m_ *′* of 0.10–0.70. (b) Mean *F*_v_ *′/F*_m_ *′* values ± SE taken before and after treatment and at regular intervals during recovery. *n* = 33–35 plants per line. Control group represent both WT and azygous plants.

Changes in CO_2_ conditions that trigger photorespiration have been associated with rapid damage to PSII, which can be measured by a decline in the *F*_v_ */F*_m_ PSII quantum yield parameter (Badger *et al*., 2009; South *et al*., 2017; Takahashi *et al*., 2007). Based on this, it was hypothesized that the improved photosynthesis observed in the ST‐LS1::H OX plants may be due to protection of PSII from damage. To test this, ST‐LS1::H OX lines G04 and G46, used in the 2017 field study, were grown for 9 days under controlled conditions (23 °C day, 18 °C night, 16‐h light, 8‐h dark cycle) and then transferred for 24 h to high photorespiratory conditions of low CO_2_ and continuous light. Following this treatment, the plants were returned to the original growth conditions and the efficiency of PSII was monitored for 24 h using the *F*_v_ *′/F*_m_ *′* parameter. The results show that the ST‐LS1::H OX plants displayed consistently higher PSII efficiency after the treatment, as indicated by higher *F*_v_ *′/F*_m_ *′* values throughout the recovery period (Figure 6).

## Discussion

In this study, we explored the impact of expressing *Arabidopsis* H‐protein in tobacco plants grown under glasshouse and field conditions. We describe the production and analysis of these overexpressor lines, followed by the testing of the ST‐LS1::H OX plants in glasshouse conditions in the UK and under field conditions for two consecutive years in mid‐west USA. Importantly, this analysis revealed increases in the biomass of these transgenic plants under both of these environments and highlighted the potential that this manipulation has for improving yields of field‐grown crops.

Although increased photosynthetic rates were observed in the 2017 field trials, we did not observe a consistent increase in photosynthesis for all lines, in all experiments. Given these results, it is possible that the increased amount of H‐protein is affecting other processes which might contribute to the observed gains in biomass. It has been described previously that when plants are transferred from high to low CO_2_ concentrations, creating high photorespiratory conditions, there is a rapid inhibition of photosynthetic CO_2_ fixation, and associated damage to PSII. This effect on PSII can be observed as a decrease in the CF parameter, *F*_v_/*F*_m_ (Badger *et al*., 2009; Takahashi *et al*., 2007). Analysis of the ST‐LS1::H OX plants after exposure to low CO_2_ conditions provides evidence that these plants are able to cope better with high photorespiratory conditions. This result may explain the observed improvements in photosynthesis and growth especially under higher light and higher temperature conditions when photorespiratory stress may be increased. This is particularly relevant under field conditions where higher temperatures and light levels (compared to glasshouse) would favour plants with a better ability to recover from photorespiratory stress damage and lead to a greater accumulation of biomass over the course of the growing season.

In search of the greatest impact on yield, we used two constructs to achieve a range of H‐protein overexpression. To achieve high expression levels, we used the CaMV 35S viral promoter which has been widely and successfully used in the past to drive high expression of the desired transgene and in the production of high yielding plants (Kohler *et al*., 2017; Lefebvre *et al*., 2005; Simkin *et al*., 2015). Additionally, the ST‐LS1 promoter, previously used for the overexpression of GCS proteins in *A. thaliana* (Timm *et al*., 2012, 2015, 2017), allowed us to achieve an intermediate level of overexpression in leaves. Somewhat unexpectedly however, the two sets of transgenic plants had contrasting growth phenotypes, and the transgenic plants with the larger increases in H‐protein had a severe slow growth phenotype. This negative effect on early growth was not explained by reduced photosynthetic rates, but interestingly, analysis of the carbohydrate profiles revealed a change in the allocation of carbon between soluble carbohydrates and starch. In the 35S::H OX plants, a 23%–59% decrease in the measured soluble carbohydrates was observed while starch accumulation increased. It has been shown previously that imbalances in carbohydrates can affect growth (Smith and Stitt, 2007; Stitt and Zeeman, 2012; Sulpice *et al*., 2009; Tognetti *et al*., 2013; Vonschaewen *et al*., 1990). Specifically, research with *A. thaliana* accession lines has also revealed a negative correlation between biomass and steady state starch levels. These studies have shown how accessions with the lowest investment in starch display the fastest growth (Sulpice *et al*., 2009). Our hypothesis that growth in the 35S::H OX is affected by starch accumulation due to inability to break it down to fuel growth during the night, is reinforced by studies of slow growing mutants (e.g. *sex1* or the *mex1*). These mutants accumulate transitory starch by failing to fully degrade it at night (Caspar *et al*., 1991; Niittyla *et al*., 2004; Streb and Zeeman, 2012; Yu *et al*., 2001). It is likely then that lower amounts of soluble carbohydrates together with the accumulation of starch are significantly impacting on the growth of the 35S::H OX plants.

Lipoylation is a post‐translational modification essential for the activity of a number of multimeric metabolic complexes including the GCS, through the H‐protein (Douce *et al*., 2001), and the E2 subunits of the enzymatic complexes PDH and KDH which are critical in regulating distinct carbon entry points into the central metabolic pathway of the tricarboxylic acid cycle (Rowland *et al*., 2018). In both the ST‐LS1::H and 35S:::H OX plants, the lipoylation signal corresponding to the H‐protein is increased, which is consistent with an increased amount of this protein in the leaves of these plants. However, the relative signal intensities for H‐protein with LA and H antibodies in the protein leaf extracts of ST‐LS1::H and 35S:::H OX plants do not match, as there is a similar increase in the signal for LA, but a considerable threefold sevenfold increase in H‐protein for 35S::H OX compared to the ST‐LS1::H. This suggests that we have a greater increase in H‐protein than of the lipoylated form of this protein in the 35S::H OX plants. If this is the case, and the H‐protein pool in the H‐protein OX plants is incompletely lipoylated, it would be expected that the activity of the GCS would be decreased. This would then affect both the role of this system in photorespiration in leaves, and in C1 metabolism in a range of tissues by disturbing the tetrahydrofolate pools. Furthermore, reduced activity of the GCS would compromise photorespiration and lead to 2PG accumulation. As 2PG levels have been proposed to have a role in regulating central carbon metabolism, including starch synthesis (Flugel *et al*., 2017), it could be expected that these transgenics would present changes in the carbohydrate metabolism, like the observed increases in starch.

Comparisons of the lipoylation of root proteins between the 35S::H, ST‐LS1::H OX and control plants revealed an increase in the amount of the H‐protein only in the 35S::H OX plants. In addition, changes in the lipoylation of root PDH and KGDH were also observed in 35S::H OX plants. It has been described that the WT amounts of H‐protein in nonphotosynthetic tissues is much lower than in photosynthetic tissues (Douce *et al*., 2001; Mouillon *et al*., 1999). It is therefore possible that the increased amount H‐protein in the roots of the 35S::H OX plants is sequestering the available LA or octanoyl moieties, leading to the observed reduction in the lipoylation signal for PDH and KGDH in the roots of these lines. At this stage, we cannot dismiss the possibility that the reduced lipoylation signal in PDH and KGDH in roots of 35S::H OX plants is due to reduced amounts of the proteins in these plants; mutant studies in *A. thaliana* plants with reduced amounts of the E2 form of PDH have shown how lack of this protein compromises plant growth (Song and Liu, 2015; Zhang *et al*., 2012). But it is interesting to note that the 35S:H OX slow growth phenotype and reduced amounts of lipoylated PDH and KGDH E2 proteins in the roots matches that of *A. thaliana* plants with reduced levels of lipoate‐protein ligase essential for mitochondrial protein lipoylation (Ewald *et al*., 2014). Taken together, these results provide evidence that it is possible that the reduced amounts of lipoylation of these proteins in the roots is linked to the slow growth phenotype.

To conclude, we have shown that increases in the amount of H‐protein in tobacco can impact on carbohydrate allocation, protein lipoylation, photosynthesis and growth, and can lead to gains in biomass under both controlled and field conditions. The ST‐LS1::H OX plants highlight the potential of this particular manipulation for the improvement of biomass and yield in other crop species, in an area of metabolism which has been largely unexploited. This is of relevance in this period of climate change, where rising annual temperatures are beginning to impact crop yields and consequently food security and biofuel production. There is a need to engineer new varieties that are not only higher yielding but which are able to sustain those higher yields under a variety of environmental conditions; optimization of photorespiration might be an important target to consider (Timm *et al*., 2016). Furthermore, there is evidence from work on *A. thaliana* that combining H‐protein overexpression with improved CB cycle function (via SBPase and FBPA overexpression) further increases biomass and seed yield (Simkin *et al*., 2017). These data raise the possibility that by combining overexpression of the H‐protein with other traits shown to improve photosynthesis and yield under field conditions, such as the simultaneous overexpression of violaxanthin de‐epoxidase (VDE), PSII subunit S (PsbS) and zeaxanthin epoxidase (ZEP) for optimization of the NPQ mechanism (Kromdijk *et al*., 2016) or the expression of the cyanobacterial bifunctional fructose‐1,6/sedoheptulose‐1,7‐bisphosphatase (FBP/SBPase) (Kohler *et al*., 2017) could enable the development of resilient new varieties that are able to sustain improved yields under a range of environmental conditions.

## Experimental procedures

### Assembly of binary constructs for overexpression of H‐protein

The coding sequence for gene AT2G35370 from *A. thaliana*, the promoter sequence of ST‐LS1 from potato (Stockhaus *et al*., 1989) and the HSP terminator (Nagaya *et al*., 2010) were synthesized for Golden Gate cloning (Engler *et al*., 2008, 2009) and cloned into Level 0 (L0) vectors. Restriction enzyme digestion of the Level 0 modules and T4‐ligase directed ligation into a Level 1 (L1) plasmid yielded the transcriptional unit pST‐LS1:AtH‐protein:tHSP. The same procedure but instead using the constitutive promoter from cauliflower mosaic virus (*CaMV*) 35S was used to generate p35S:AtH‐protein:tHSP. These L1 transcriptional units were assembled downstream of the plant selection marker, *HptII* (hygromycin B) with the CaMV 35S promoter and the CaMV 35S terminator sequences. To produce the Level 2 binary expression constructs (ST‐LS1::H and 35S::H, respectively). Additionally, the ST‐LS1::H construct also carried a bialaphos resistance gene whose expression was controlled by the nopaline synthase promoter and terminator.

### Generation of transgenic plants by Agrobacterium‐mediated transformation

Wild‐type tobacco (*N. tabacum* cv Petite Havana) leaf discs were transformed using *Agrobacterium tumefaciens* strain LBA4404 harbouring the binary cassettes (Horsch *et al*., 1985). Shoots were regenerated on Murashige and Skoog (MS)‐based medium containing hygromycin (20 mg/L), cefotaxime (400 mg/L), 6‐benzylaminopurine (BAP; 1 mg/L) and 1‐naphthaleneacetic acid (NAA; at 0.1 mg/L) for 2 weeks. Thereafter, the leaf discs were transferred to medium containing increased concentration of BAP (2.5 mg/L) and also indole‐3‐acetic acid (IAA; 0.2 mg/L) in a protocol modified from Pathi *et al*. (2013). Juvenile shoots derived from somatic embryos were then isolated and cultivated on MS‐based media with antibiotic selection until roots developed. Putative transformants were transferred to soil and used for molecular analysis. Transformations were incubated in growth chambers under a 16 h light, 22 °C and light intensities between 130 and 300 μmol/m^2^/s.

All T0 rooted shoots were sampled for T‐DNA copy number and transgene expression analysis and allowed to self‐fertilize and then selected on the basis on having a single insertion event, and detectable levels of transgene mRNA and protein. T1 plants were grown in soil, sampled (for copy number and transgene expression analysis) and allowed to self‐fertilize. Azygous (also known as null segregants), and transgenic segregants (homozygous when possible) were selected at this stage based on protein expression and used for T2 growth analysis. Control plants throughout the paper represent a mix of WT and azygous segregants from the transgenic lines selected, verified by PCR for lack of the transgene and absence of overexpression by qRT‐PCR and immunoblot. qPCR analysis used for estimation of T‐DNA copy number (as number of *HptII* copies) and zygosity in T0, T1 and T2 was carried out by IDNA Genetics Ltd (Norwich Research Park, UK) in a similar manner as reported previously (Bartlett *et al*., 2008). A summary table of the segregation results is presented in Table S1.

### Protein extractions and immunoblotting

Leaf discs were ground in dry ice and protein extractions performed as described in Lopez‐Calcagno *et al*. (2017) and using the nucleospin RNA/Protein kit (Macherey‐Nagel, http://www.mn-net.com/). Protein quantification was performed using the protein quantification Kit from Macherey‐Nagel. Samples were loaded on an equal protein basis (8–0.1 μg per well depending on the experiment), separated using 12% (w/v) SDS‐PAGE, transferred to nitrocellulose membranes, and probed using antibodies raised against each of the GDC complex proteins (Timm *et al*., 2012) and LA (Merck KGaA, Darmstadt, Germany). Proteins of interest were detected using horseradish peroxidase conjugated to the secondary antibody and ECL chemiluminescence detection reagent (Amersham, Buckinghamshire, UK). In addition to the aforementioned antibodies, samples were probed using antibodies raised against transketolase (Henkes *et al*., 2001) and actin (Agrisera, via Newmarket Scientific, UK).

### Plant growth conditions

#### Controlled conditions experiments

T2 seeds (homozygous and segregating) were germinated on soil in controlled environment chambers at an irradiance of 130 μmol/m^2^/s, 22 °C air temperature and relative humidity of 60%, in a 16‐h photoperiod. Two weeks after sowing, seedlings were transferred to individual 8‐cm pots and grown under the same conditions for 7–14 days. Fourteen to 20 days after sowing, leaf areas were calculated using a CF imaging system (Technologica, Colchester, UK; Baker and Rosenqvist, 2004; Barbagallo *et al*., 2003), or alternatively from photographs using ImageJ (http://rsb.info.nih.gov/ij/index.html). Twenty‐two days after sowing, plants were transferred into 4‐L pots and were moved to a controlled environment glasshouse (16‐h photoperiod, 25 °C–35 °C day/20 °C night air temperature, with natural light supplemented under low light with high‐pressure sodium light bulbs, at 380–600 μmol/m^2^/s). Position of the plants was changed daily and watered regularly with a nutrient medium (Hoagland and Arnon, 1950). Plants were positioned such that at maturity, a near‐to‐closed canopy was achieved and the temperature range was maintained similar to the ambient external environment. Six to seven weeks after sowing, when the first flower buds began to appear plants were harvested for biomass. Stem length and the number of leaves per plant were determined, and total leaf area per plant measured with a conveyor‐belt scanner (LI‐3100C Area Meter; LI‐COR, Lincoln, NE). Plants were subsequently separated into leaf and stem fractions and dried to constant weight at 70–80 °C, after which dry weight was determined for each fraction.

#### Field experiments

The field site was situated at the University of Illinois Energy Farm (40.11°N, 88.21°W, Urbana, IL). Two different experimental designs were used. For 2016: A replicated control design was implemented (Figure S6a). Plants were grown in 4 × 14 rows, with plants spaced 30 cm apart with the outer boundary of plants being a wild‐type border. The entire experiment was surrounded by two rows of wild‐type borders. The plants were irrigated with rain towers in the evening, when required. T2 seed was germinated and after 11 days were moved to individual pots (350 mL). The seedlings were grown in the glasshouse for 15 days before being moved into the field. Harvest occurred after a further 15 days.

For 2017: The experimental design was set up as blocks within rows, where 1 block holds one of each of the five constructs and each row has all lines (Figure S6b). The central 20 plants of each block are divided into five rows of four plants per genotype. Plants were arranged in an east to west orientation. T2 seed was germinated and after 12 days moved to hydronic trays (Transplant Tray GP009 6 × 12 cells; Speedling Inc., Ruskin, FL), and grown for 24 days before being moved to the field. The plants were harvested after 30 days in the field.

The field was prepared in a similar fashion each year in terms of fertilizer and how pesticides were applied [described in Kromdijk *et al*. (2016)]. Light intensity (LI‐quantum sensor; LI‐COR) and air temperature (Model 109 temperature probe; Campbell Scientific Inc, Logan, UT) were measured nearby on the same field site, and half‐hourly averages (Figure S7) were logged using a data logger (CR1000; Campbell Scientific).

### Chlorophyll fluorescence imaging

Measurements were performed on either 9‐day‐old or 3‐ to 4‐week‐old tobacco seedlings that had been grown in a controlled environment chamber under 130 μmol/mol^2^/s PPFD, and ambient CO_2_. CF parameters were obtained using a CF imaging system (Technologica; Baker and Rosenqvist, 2004; Barbagallo *et al*., 2003).

Nine‐day‐old seedlings grown in MS +3% sucrose plates were moved to a sealed clear plastic container with low CO_2_ conditions (between 0 and 30 μmol/m^2^/s) and constant illumination for 24 h. Measurements of maximum efficiency (*F*_v_′*/F*_m_′) were taken before and after treatment in a similar manner as previously described (Badger *et al*., 2009; Oxborough and Baker, 1997; South *et al*., 2017).

The operating efficiency of photosystem two (PSII) photochemistry, *F*_q_′*/F*_m_′, was measured in 3‐ to 4‐week‐old seedlings. Images of *F*′ were taken when fluorescence was stable at 200 and at 600 μmol/m^2^/s PPFD, whilst images of maximum efficiency (*F*_v_′/*F*_m_′) were obtained after a saturating 600‐ms pulse of 6200 μmol/m^2^/s PPFD and calculated as previously described (Baker *et al*., 2001; Oxborough and Baker, 1997, 2000).

### Leaf gas exchange measurements

The response of net photosynthesis (*A*) and stomatal conductance (*g*_s_) to light was measured using a portable infrared gas analyser (LI‐COR 6400XT; LI‐COR). For both experiments, light was set to 10% blue, and temperature was set to 2 °C above ambient levels, flow at 300 μmol/s and VPD at 1 (±0.2). Under glasshouse conditions, leaves were initially stabilized at saturating irradiance 2000 μmol/m^2^/s, and a reference CO_2_ of 400 μmol/m^2^/s, after which measurements were made at the following PPFD levels: 2000, 1650, 1300, 1000, 750, 500, 400, 300, 200, 150, 100, 50 and 0 μmol/m^2^/s. Measurements were recorded after *A* reached a new steady state (1–2 min). In the field, a saturating irradiance of 2200 μmol/m^2^/s was used, and PPFD levels for the light curves were as follows: 2200, 2000, 1700, 1400, 1100, 800, 600, 425, 250, 150, 100, 50 and 0 μmol/m^2^/s.

### Soluble sugar and starch determination

Plants were germinated and grown under controlled environment conditions (22 °C, 16‐h light, 8‐h dark cycle) for 20 days and then transferred to glasshouse conditions. At 4 or 5 weeks postgermination, the youngest fully expanded leaf was taken at mid‐day and snap frozen in liquid nitrogen. The tissue was then freeze‐dried, and 6 mg aliquots of the tissue were used for carbohydrate analysis. Each six mg sample was incubated in 80% (v/v) ethanol for 60 min at 80 °C and then washed two times with ethanol 80% (v/v). Sucrose, glucose and fructose were measured from the ethanol extract using an enzyme‐based protocol (Stitt and Quick, 1989), and the starch contents were estimated from the ethanol‐insoluble pellet according to Stitt *et al*. (1978).

### Statistical analysis

All statistical analyses were performed using ANOVA followed by a post hoc Tukey test using dynamic means. Analyses were carried out in SPSS (Statistical Product and Service Solutions) or R (https://www.r-project.org/)**.**

## Conflict of interest

The authors declare no conflict of interest.

## Author contributions

C.A.R. conceived this project, provided the funding and supervised the research with input from P.E.L.C. P.E.L.C. and S.E.B. designed and generated constructs for *Agrobacterium‐*mediated transformation of plant material. P.E.L.C., K.L.B. and S.E.B. generated transgenic plants. P.E.L.C. and K.L.B. performed molecular, biochemical and plant phenotypic analysis. S.F. carried out carbohydrate assays. P.F.S. adapted the low CO_2_ system, and P.E.L.C and P.F.S. collected the data. All authors carried out data analysis on their respective contributions. The manuscript was written by P.E.L.C and C.A.R. All authors reviewed and commented on the final manuscript.

## Supporting information


**Figure S1** Schematic representation of the constructs used for tobacco transformation.
**Figure S2** Expression of the GCS proteins P, L, T does not change in T2 H‐protein OX plants.
**Figure S3** Overexpression of the H‐proteins can affect lipoylation of other proteins.
**Figure S4** Overexpression of the H‐protein impacts on plant growth rate.
**Figure S5** Overexpression of the H‐protein of the glycine cleavage system increases plant growth. Additional parameters.
**Figure S6** Field experiments layouts.
**Figure S7** Environmental conditions during field growth.
**Table S1** Segregation of H OX lines in T0, T1 and T2 generation.
