# Supplementary material for: Overexpressing the H‐protein of the glycine cleavage system increases biomass yield in glasshouse and field‐grown transgenic tobacco plants
Source: Plant Biotechnol J. 2018 Jul 22;17(1):141–51. doi: 10.1111/pbi.12953 (PMC6330538; doi:10.1111/pbi.12953)
Supplement: Supplementary file 1 — Figure S1 Schematic representation of the constructs used for tobacco transformation. Figure S2 Expression of the GCS proteins P, L, T does not change in T2 H‐protein OX plants. Figure S3 Overexpression of the H‐proteins can affect lipoylation of other proteins. Figure S4 Overexpression of the H‐protein impacts on plant growth rate. Figure S5 Overexpression of the H‐protein of the glycine cleavage system increases plant growth. Additional parameters. Figure S6 Field experiments layouts. Figure S7 Environmental conditions during field growth. Table S1 Segregation of H OX lines in T0, T1 and T2 generation. [file PBI-17-141-s001.pdf]

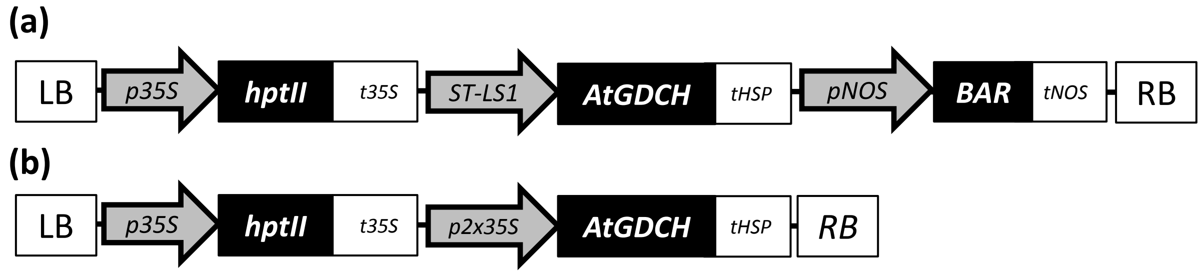

Supplemental Figure S1.- Schematic representation of the constructs used for tobacco transformation

(a) Construct used to produce the ST-LS1::H OX lines **(b)** Construct used to produce the 35S::H OX lines**.** LB, T-DNA left border; *p35S*, cauliflower mosaic virus 35S promoter; *hptII*, hygromycin phosphotransferase gene; *t35S*, cauliflower mosaic virus 35S terminator; *ST-LS1*, *Solanum tuberosum ST-LS1* promoter; *AtGDCH*, *Arabidopsis thaliana* cDNA for the H-protein; *tHSP,* heat shock protein 18.1 terminator*; pNOS*, nopaline synthase promoter; *BAR*, Bialaphos resistance gene; *tNOS*, nopaline synthase terminator; RB, T-DNA right border.

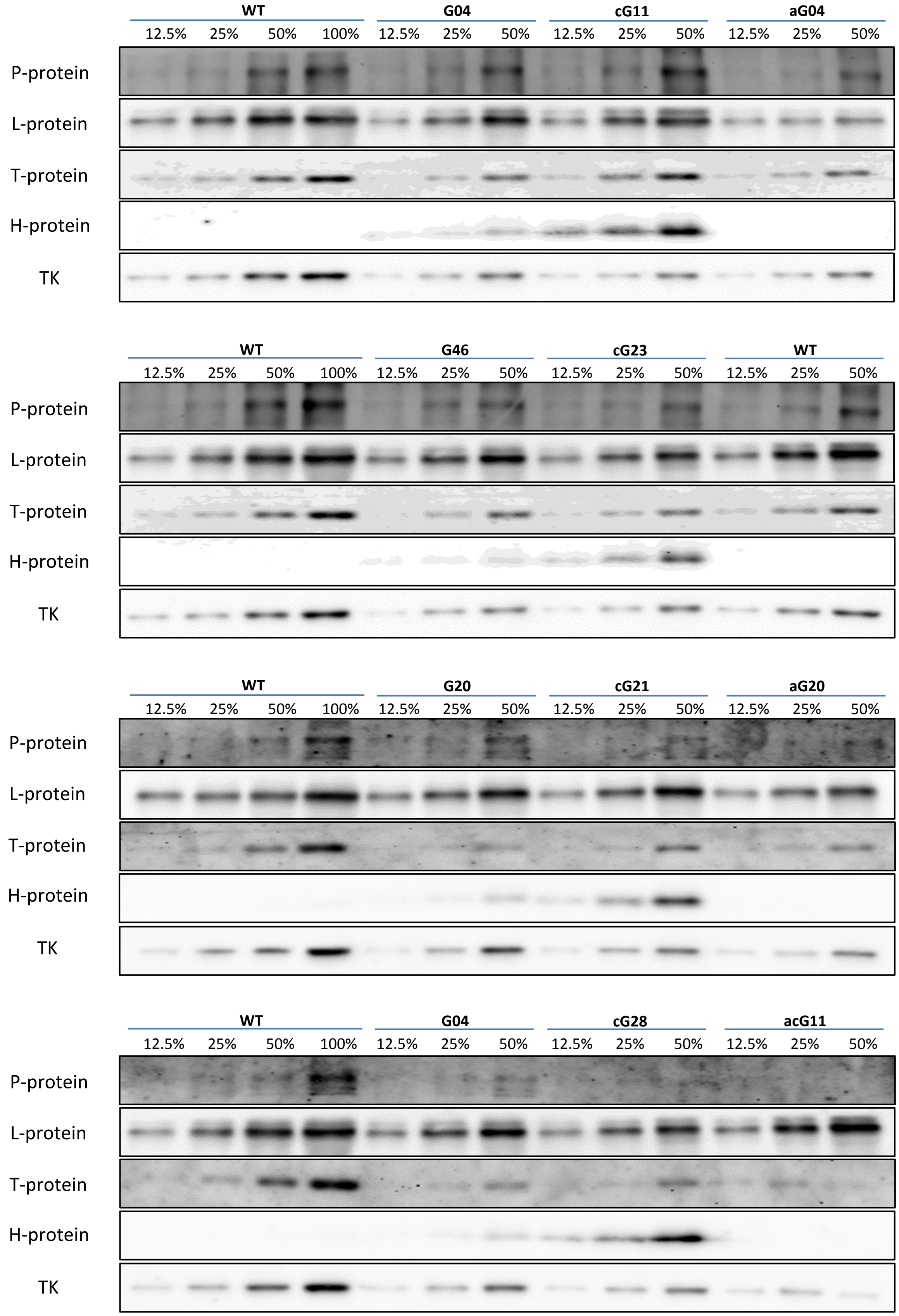

Supplemental Figure S2.- Expression of the GCS proteins P, L, T does not change in T2 H-protein OX plants.

Serial dilutions of crude leaf proteins were loaded for each line, and blotted against each of the proteins of the glycine cleavage system and TK (transketolase). ST-LS1::H OX are designated with the letter G (G04, G20 and G46), 35S::H OX are designated with the letters cG (cG11, cG21, cG23 and cG28) and controls are represented by WT plants and azygous plants (designated by the "a" prefix and followed by the code from the line they segregated from: aG04, aG20 and acG11). 100% corresponds to 0.5 µg of crude protein extract. TK was used as loading control.

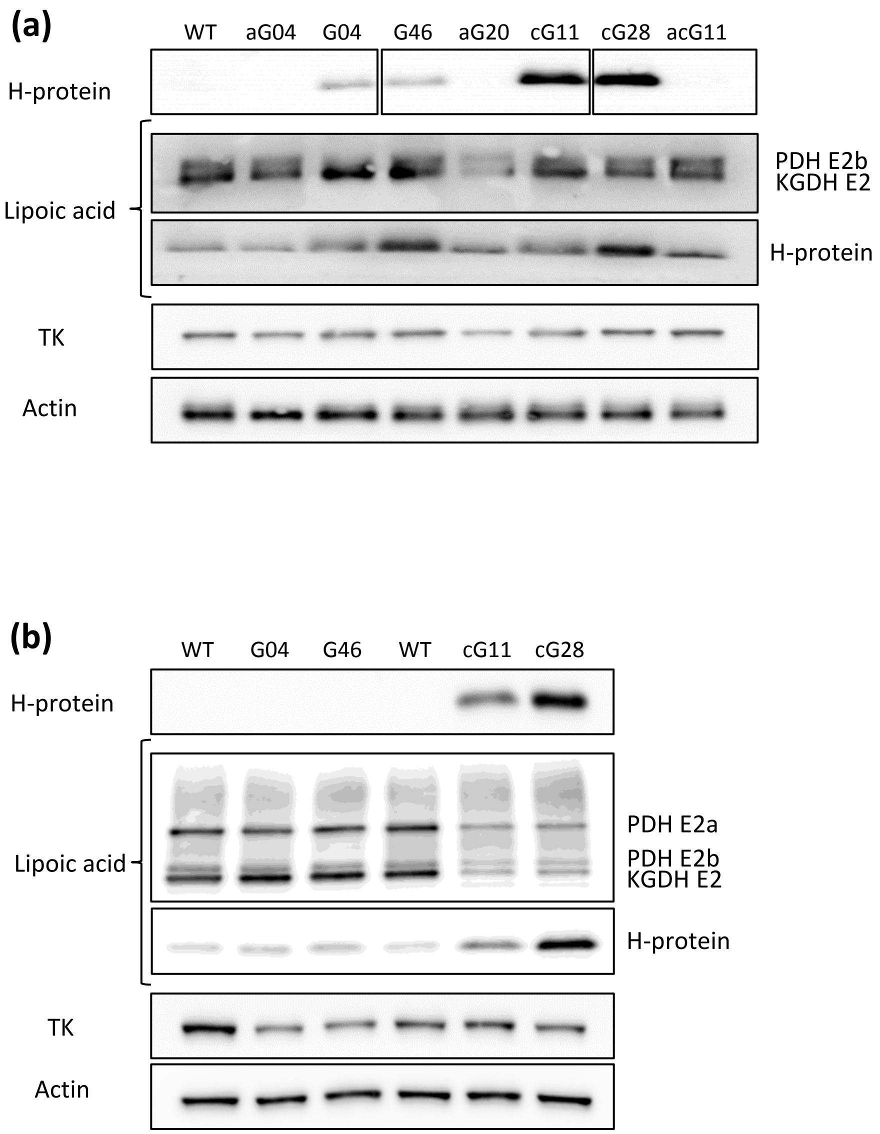

Supplemental Figure S3.- Overexpression of the H-proteins can affect lipoylation of other proteins.

Crude protein extracts from leaf (1µg) and roots (0.1µg) were loaded per well and blotted against H-protein, Actin, TK and lipoic acid. **(a)** Leaf extracts, **(b)** root extracts. ST-LS1::H OX are designated with the letter G (G04 and G46), 35S::H OX are designated with the letters cG (cG11 and cG28) and controls are represented by WT and azygous plants (designated by the "a" prefix and followed by the code from the line they segregated from: aG04, aG20 and acG11). Actin and TK were used as loading controls. PDH-E2 variants were named as described by Taylor et al. (2002).

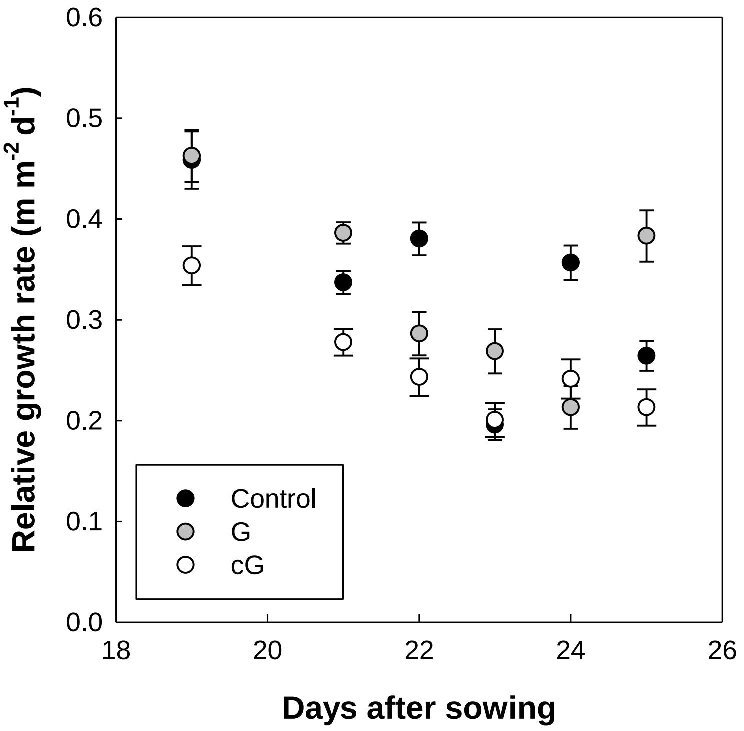

RGR (m m^{-2} d^{-1}) ± SE

Days after sowing	Control	G	cG
19	0.458 ± 0.028	0.462 ± 0.026	**0.354 ± 0.019***
21	0.337 ± 0.011	**0.386 ± 0.011***	**0.278 ± 0.013****
22	0.380 ± 0.016	0.286 ± 0.022	**0.243 ± 0.019*****
23	0.196 ± 0.015	**0.269 ± 0.022***	0.201 ± 0.017
24	0.357 ± 0.017	**0.213 ± 0.021*****	**0.241 ± 0.019*****
25	0.264 ± 0.015	**0.383 ± 0.025***	0.213 ± 0.018

Supplemental Figure S4.- Overexpression of the H-protein impacts on plant growth rate.

Relative growth rate as leaf area vs time (RGR= $(\ln(LA_f)-\ln(LA_i))/(t_f-t_i)$) over an eight day period, for plants germinated and grown in a controlled environment growth room (22°C, 16 h light, 8 h dark cycle) for 25 days. Control group (WT and azygous; black circles), ST-LS1::H OX (G; grey circles), 35S::H OX (cG; white circles). Mean values ± SE are indicated, n=5-12 plants per line, Control group represent both WT and azygous plants. Asterisks indicate significance between OX lines and control group using ANOVA with Tukey's post-hoc test, *p<0.05, **p<0.0005, ***p<0.00005.

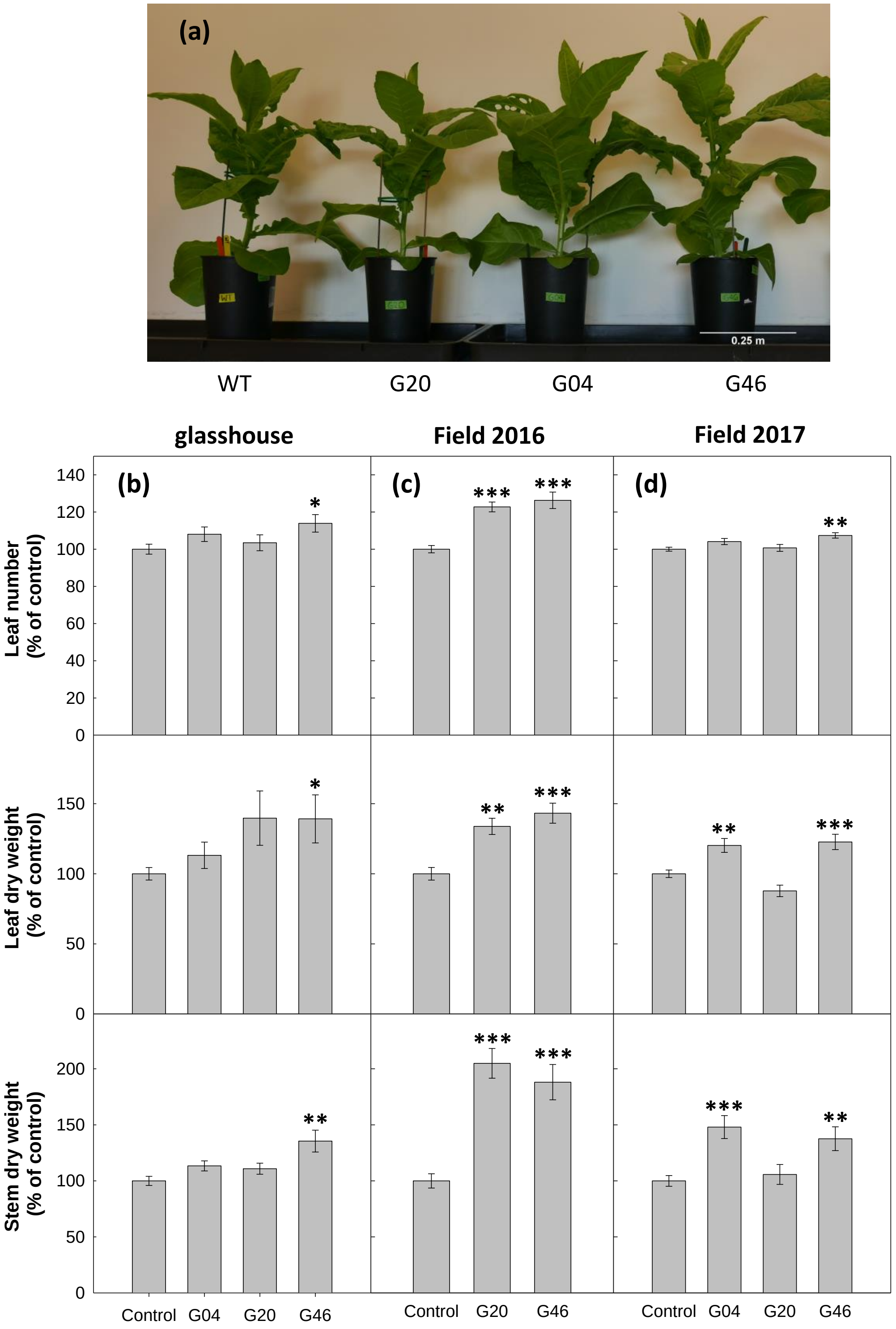

Supplemental Figure S5.- Overexpression of the H-protein of the glycine cleavage system increases plant growth. Additional parameters

(a) Appearance of 40 day old glasshouse-gown tobacco plants. **(b)** 40 day old glasshouse-grown tobacco plants **(c)** 40 day old 2016 field-grown plants. (Plants were germinated and grown in glasshouse conditions for 26 days and then allowed to grow in the field in summer 2016 for 14 days) **(d)** 56 day old 2017 field-grown plants. (Plants were germinated and grown in glasshouse conditions for 26 days and then allowed to grow in the field in summer 2017 for 30 days). Harvest data; number of leaves, leaf dry weight, and stem dry weight are displayed. Control group represent both WT and azygous plants. Mean +/- SE presented. Plants per line: **(b)** n= 4-6, **(c)** n=6, **(d)** n= 24. Asterisks indicate significance between OX lines and control group using ANOVA with Tukey's post-hoc test, *$p<0.05$, **$p<0.005$, ***$p<0.0005$

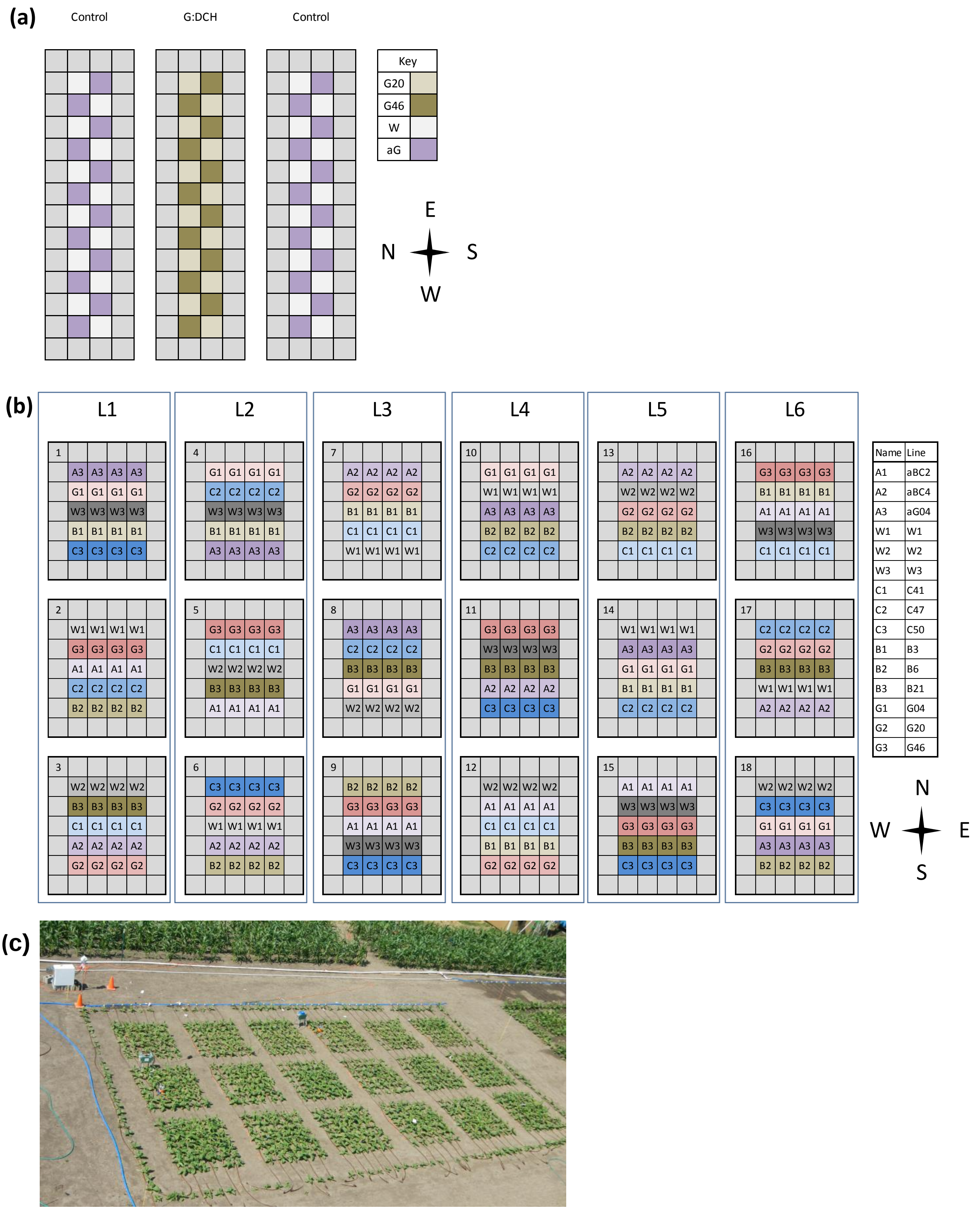

Supplemental Figure S6. Field experiments layouts

Schematic to represent the experimental field designs for 2016 and 2017. (**A**) 2016 Field experiment were designed to a replicated control design. Each row was 4x14 with the outer plant being a wild type border control. Two lines per row were planted in an alternating pattern. The controls for each transgenic construct were the two rows of controls either side of the transgenic row. (**B**) 2017 field design was randomised rows split into three blocks, where one row has all 15 lines and one block had one line of each construct. Rows were randomised using RAND function, Microsoft Excel 2010. Both experiments were surrounded by a wild type border and each separate experiment was bordered by two lines of wild type. (**C**) Picture of 2017 field trial day before harvest.

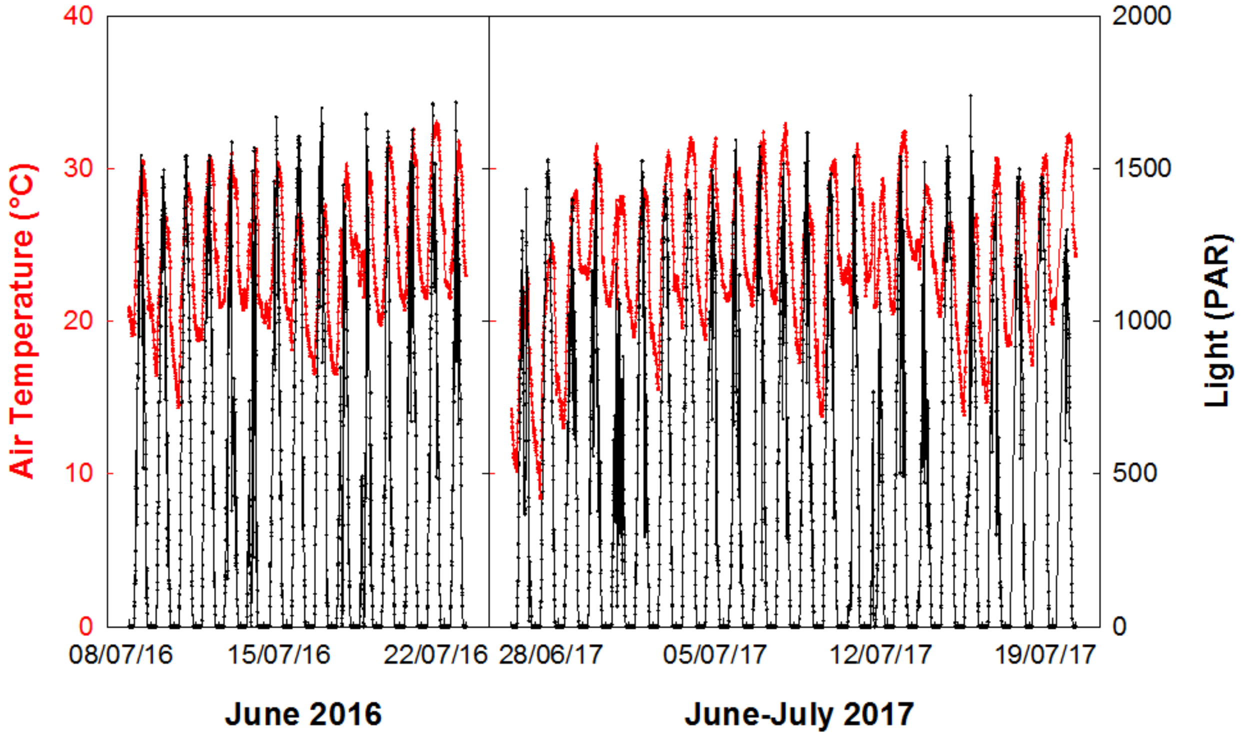

Supplemental Figure S7. Environmental conditions during field growth

Air Temperature (˚C) and light intensity (PAR) from 2016 and 2017 growth seasons. Data were collated into 30 min averages

Construct	Line	T0 (qPCR determined insert number for the event selected)	T1 (Proportion AZ:HZ:HM)	T2 (Percentage of transgenic plants)
ST-LS1::H	**G04**	1	3:5:1	100%
	**G20**	1	3:5:2	100%
	**G46**	1	0:5:5	100%
35S::H	**cG11**	1	6:2:0	31%
	**cG21**	1	4:6:0	30%
	**cG23**	1	3:7:0	57%
	**cG28**	1	2:7:1	100%

Supplemental Table S1.- Segregation of H OX lines in T0, T1 and T2 generation

qPCR-determined insert number for selected T0 lines and segregation on T1 as a proportion of: null segregants (AZ, no T-DNA), hemizygous (HZ, one T-DNA copy) and homozygous (HM, two T-DNA copies) plants. Whenever possible, HM plants were taken forward to T2, with the exception of azygous control lines aG04 and aG20 where null segregants were also taken forward, and of cG11, cG21 and cG23 where no HM plants were identified. The segregation state of these lines at the T2 generation was confirmed by PCR or qPCR analysis, the percentage of PCR positive plants is presented. A minimum of 8-10 plans were analysed for segregation in T1 and T2 generations.
